# Ovarian tissue cryopreservation for fertility preservation: a two-decade single-center experience with 451 children and adolescents

**DOI:** 10.1186/s12958-025-01388-x

**Published:** 2025-04-05

**Authors:** Norah L. A. Emrich, Rebekka Einenkel, Cara Maria Färber, Andreas Schallmoser, Nicole Sänger

**Affiliations:** Department of Gynecological Endocrinology and Reproductive Medicine, University Hospital of Bonn, Venusberg Campus 1, Bonn, 53127 Germany

**Keywords:** Fertility preservation, Ovarian tissue cryopreservation, Children, Adolescents, Vitrification, Storage duration, Storage behavior, Gonadotoxic treatment, Primary ovarian insufficiency, Mortality rate

## Abstract

**Background:**

Ovarian tissue cryopreservation (OTC) is the only fertility preservation option for premenarcheal girls before gonadotoxic treatment, but is still considered to be experimental in pediatric patients. This study investigated storage behaviors across different age groups to refine counseling approaches for pediatric patients.

**Methods:**

This retrospective study analyzed data from children (0–14 years), adolescents (15–19 years), and adults (≥ 20 years) who underwent OTC between 2000–2021 at the University Hospital Bonn's cryobank. Comparison to adults (age ≥ 20 years) was conducted.

**Results:**

Of 2,475 patients, 6% were children and 12% adolescents. Sarcoma was most common in children, lymphoma in adolescents. Adults had longer active storage than children (5.5 vs. 4.7 years, *p* = 0.011), but for active storage ≥ 10 years, children and adolescents stored longer than adults (13.1 and 12.6 vs. 11.8 years, p ≤ 0.01). The proportion of adolescents increased, while that of children decreased in long-term storage. Median ovarian cortex surface before cryopreservation was 3.5 cm^2^ in children and 4.5 cm^2^ in adolescents. Leukemia and sarcoma had the highest mortality rates in children (25% and 13.5%). Overall, pregnancy and birth rates following ovarian tissue transplantation (OTT) were 34.5% and 24.1%, respectively. Among adolescents, pregnancy rates were 33.3% after OTT and 27.3% without OTT, while all children without OTT achieved pregnancy (100%).

**Conclusions:**

Children and adolescents represent a small subset of OTC patients, with indications linked to common pediatric malignancies. For active storage ≥ 10 years, they store longer than adults, likely due to delayed reproduction or awaiting in vitro growth / in vitro maturation in hematological cases. Overall, adults store longer, but adolescent storage has risen over time possibly due to higher child mortality and previously limited OTC use in younger patients. Mean ovarian cortex surface data may guide pediatric tissue harvest recommendations, with unilateral oophorectomy advised. Fertility preservation counseling and cost coverage should be standard for pediatric patients undergoing gonadotoxic treatment. A tailored approach to OTC indications is essential, especially in high-mortality cancers like leukemia or sarcoma. Favorable pregnancy rates observed, even without OTT, suggest possible OTC overutilization, highlighting the need for individualized strategies and careful clinical decision-making to balance risks and preserve reproductive potential.

## Background

Thirty-five thousand new cases of cancer in children and adolescents are diagnosed every year in Europe [1], 2,250 of these are newly diagnosed cases in Germany [2]. However, survival rates in pediatric oncology are fortunately constantly increasing due to advances in systemic and locoregional treatment. The overall 5-year survival rate in childhood cancers is approximately 80% [3–5]. Therefore, particular attention should be paid at life quality of cancer survivors [4, 6]. One of the most important subjects after a successfully treated oncologic disease is family planning. Of those men and women having survived cancer, 76% wish to have a child [7, 8]. However, this can be difficult due to gonadotoxic effects of most chemotherapy regimens as well as radiotherapy [8–10]. Only 12% of oncologic patients feel adequately informed about fertility preserving measures before receiving gonadotoxic treatment [7, 8]. In prepubescent girls, it is assumed that the only feasible option of fertility preservation is ovarian tissue cryopreservation (OTC). While the majority of literature supports the notion that producing mature gametes is not feasible in prepubertal girls, a limited number of studies have demonstrated the potential for oocyte retrieval following ovarian stimulation in this demographic [8, 9, 11–13]. Through performing OTC, primordial follicles are preserved embedded in ovarian cortex [11].

In adults, OTC is an already established method with the first published childbirth after transplantation of ovarian tissue (OTT) in 2004 [14]. It can be conducted without causing a time delay, which is important for patients requiring urgent initiation of chemotherapy or radiotherapy. In 2019, the American Society of Reproductive Medicine (ASRM) officially classified OTC as a non-experimental method for fertility preservation in adult patients [15]. Pregnancy rates after OTC/OTT range between 28–50% [16–19]. So far, more than 140 live births after OTC and OTT have been documented [20]. The efficacy of this fertility preservation method is well-demonstrated in adults but the data are scarce for children. OTC in pediatric patients is mostly considered to be investigational [19, 21]. The group of PanCareLIFE Consortium moderately recommends OTC also in prepubertal patients as standard care since its benefits outweigh the potential harms in the patient group with expected high gonadotoxicity [22]. However, the first live birth after performing OTC [23] in a girl before menarche with a reimplantation at her age of 27 years was achieved 2014 [24].

Post-pubertal fertility preservation options extend beyond OTC. Gonadotropin-releasing hormone agonists (GnRHa) can be used as adjuncts, inducing reversible ovarian suppression during chemotherapy through pituitary downregulation. While both direct and indirect mechanisms contribute to GnRHa's assumed protective effect, its efficacy varies due to heterogeneous data [8, 24–27]. For high gonadotoxicity cases, ovarian stimulation and oocyte vitrification are possible in post-menarcheal adolescents, potentially combined with GnRHa and OTC. However, this approach poses several challenges, including a delay of 10–14 days, the off-label use of gonadotropins in individuals under 18 years of age, and the potential for psychological or physical discomfort associated with vaginal access in cases of virgo intacta [8].

Despite its many advantages, a noteworthy obstacle of the OTC/OTT method is the possible risk of retransfer of malignant cells to the patient through OTT and the consequent possible reinduction of the oncological disease, depending on the subtype of cancer [19, 28, 29].

The University Hospital of Bonn is one of more than 150 participating clinics in Germany, Austria and Switzerland that contribute data on fertility preservation to the *Ferti*PROTEKT network, which was founded in 2006 [19, 30].

OTC is the only fertility preservation option available for prepubertal girls, and its growing evidence and demand highlight its importance. However, it presents unique challenges compared to adult patients due to the distinct storage capacities and requirements involved. Pediatric ovaries are smaller, providing less tissue for retrieval, and their ovarian cortex contains a higher density of primordial follicles but also a greater proportion of abnormal ones. These factors can influence tissue viability, functionality, and the success of transplantation or fertility restoration. The smaller size of pediatric ovarian tissue necessitates meticulous surgical techniques and specialized cryopreservation protocols to preserve viability. Additionally, long-term storage introduces logistical challenges, including maintaining tissue integrity over extended periods and ensuring accessibility when needed [31–33].

The aim of this study was to evaluate all cases of OTC and cryostorage in children (ages 0–14 years) and adolescents (ages 15–19 years) treated at the University Hospital Bonn Cryobank between 2000 and 2021. Particular focus was placed on return, pregnancy, and birth rates, as well as active storage durations and cancer subtypes, to enhance counseling strategies for pediatric patients.

## Methods and material

### Study population and storage groups

All participants enrolled in this retrospective study were either children (age 0–14 years) or adolescents (age 15–19 years). Comparison to adults (age ≥ 20 years) was conducted. The definitions of the groups by mentioned age limits are already described elsewhere [4, 34]. All patients who stored ovarian tissue in the cryobank of the University Hospital Bonn were included in this study and gave written informed consent. Patients were assessed and risk stratified for fertility issues according to national guidelines. All patients received a comprehensive counseling session. Inclusion criterion was female gender. Exclusion criteria were male gender, distant metastases and significantly reduced probability of survival [8, 30]. Data extraction was performed for all cases between 2000 and 2021 from cryobank of the University Hospital Bonn.

Active storage group was defined as patients storing their ovarian tissue in our cryobank with active current contracts and ongoing annual payments of storage fees for cryopreserved tissue samples. The two subgroups active storage group ≥ 5 years and active storage group ≥ 10 years contained patients with an active cryostorage contract and annual payments for 5 years or more or 10 years or more, respectively.

End of storage required active termination by the patient via official patient declaration stating the intention to end sample storage. Other reasons for storage end were patient’s decease, transplantation on site and outsourcing. To enable the destruction of samples following the patient’s death, death certificates were obtained. Orthotopic tissue transplantation was conducted by specially trained reproductive physicians via laparoscopy. Outsourcing comprised transfer to other cooperating centers for either OTT or further storage. OTT on site was defined as internal return rate and outsourcing for scheduled external OTT was considered external return rate. Those two combined was regarded as overall return rate. Apart from these reasons, end of storage could be intended by repeated severe violation of the contractual conditions. This was the case, for example, when patients repeatedly failed to pay and could not be contacted. If necessary, the legal department was involved in those cases. In the time period until 2023, patients in Germany had to bear the costs for OTC and storage themselves.

### Ethical approval

The procedures of this study were approved by the ethics committee of the University Hospital Bonn, Germany, approval code 007/09. In order to obtain patient’s information aside from storage and disposal data in our storage database, a specialized questionnaire was conducted. Patients gave written informed consent prior to contact via letter, e-mail or telephone. For patients who were minors, parents or legal guardians provided their written consent. After the children and adolescents reached adulthood, they were required to re-sign the cryopreservation contracts. All performed procedures were in accordance with the Helsinki Declaration.

### Tissue procedures

Ovarian tissue harvesting was performed laparoscopically either at the University Hospital Bonn or corresponding centers. In most instances, these procedures were conducted independently as standalone surgeries by different surgeons. In children, preferably one whole ovary was taken out in order to gain enough material to cryoconserve. In adults and postpubescent adolescents, approximately 50% of one ovary was excised [19]. Immediately after surgical removal of the ovary, the tissue was secured in a tube with custodiol medium (Dr. Köhler, Bensheim, Germany) and stored at 4° C until preparation. One small piece of ovarian tissue was obtained separately, inserted in formalin solution and examined histologically to exclude the presence of metastases. Ovarian tissue transplantation (OTT) was carried out laparoscopically, with the ovarian tissue being implanted into a peritoneal pocket.

In Germany, ovarian tissue from external clinics was primarily transported to three centralized cryobank facilities – University Hospital Düsseldorf, University Hospital Erlangen, and University Hospital Bonn – where it was cryopreserved and stored. Transportation of ovarian tissue from cooperating centers to cryobank of the University Hospital Bonn was facilitated by specially assembled boxes for save transportation. These custom-made metal gear boxes contained a vessel with custodiol tissue maintenance media, cold packs (delta T, Fernwald, Germany), certified temperature data loggers (delta T, Fernwald) and temperature isolating inlays (Consarctic, Westerngrund, Germany). Receiving these boxes after harvesting ovarian tissue in cooperating centers was either via overnight shipment, receiving the tissue samples one day after surgery, or non-overnight transportation on the operation day [35].

Tissue preparation was performed according to published protocols of Schallmoser et al. [36, 37]. Initially, the medulla was carefully ablated to ensure precise removal. Subsequently, the cortical tissue was sectioned into uniform strips measuring 8 × 4 × 1 mm under sterile conditions. In 2019, the ratio of the stripes was reduced to 5 mm × 4 mm × 1 mm prior to cryopreservation. Viability staining of two pieces of ovarian cortex samples of 2 mm in diameter was conducted after digestion with collagenase. Afterwards, OTC was performed by slow freezing [38] or vitrification [36, 39, 40]. Storage of the ovarian tissue was conducted in vapour phase of liquid nitrogen at −160 °C in automatically replenished storage tanks (MVEHeco Chart, Ball Ground, USA), which were guarded by an autonomous high end alarm system (Planer limited, Middlesex, GB).

### Patient data and statistical analysis

Patient data like name, birthdate, date of surgery and initial diagnosis, as well as informed consent and storage contract were collected in external as well as internal patients and enrolled in the specialized in-vitro fertilization management program MedITEX (©CRITEX, Regensburg, Germany). Furthermore, number of cortex samples, number of aliquots and storage depot parameters were entered into the database. All patient cases from 2000 to 2021 were revised, manually digitalized and entered into MedITEX (©CRITEX, Regensburg, Germany) system. Statistical analysis was performed using SPSS version 27.0 (IBM SPSS Statistics for Windows, IBM Corp., Chicago, IL, USA). Regarding continuous data, Mann–Whitney U-test was used as appropriate. Patient’s indication for OTC was divided into the following eight groups: Haematological malignancies, tumors of brain and nervous system, sarcoma, gynecological tumors (consisting of epithelial ovarian carcinoma, germ cell tumors, granulosa cell tumors, borderline tumors, squamous cell carcinoma of the cervix, adenocarcinoma of the cervix, hydatidiform mole, chorionic carcinoma, vulvar carcinoma), breast cancer, lymphoma, others (consisting of Turner syndrome, galactosemia, sickle cell disease, thalassemia major, medullar aplasia, Kostmann syndrome), and non specified (when the surgery was performed at an external facility, and no information regarding the disease subtype was available). For cancer subtypes with a high risk of reintroducing malignant cells through OTT, patients and their parents were informed that the only feasible option for utilizing the cryopreserved ovarian tissue would be in-vitro growth (IVG) and in-vitro maturation (IVM), techniques that remain experimental at this time.

### Storage fees

Based on the 3 leading ovarian tissue cryobanks in Germany annual cryopreservation fees for ovarian tissue were averaged, the result was 330.70 € per year. For the usually extraordinarily long storage times of 20 to 30 years in children and adolescents, the total storage fees would result in 6,614 € to 9,921 €, respectively. Since 2023 there is a reimbursement of costs in Germany, but only for patients after menarche, not for patients before puberty. Hormonal stimulation and cryopreservation of unfertilized oocytes are covered by insurance since July 2021 in Germany.

## Results

**Fig. 1:**
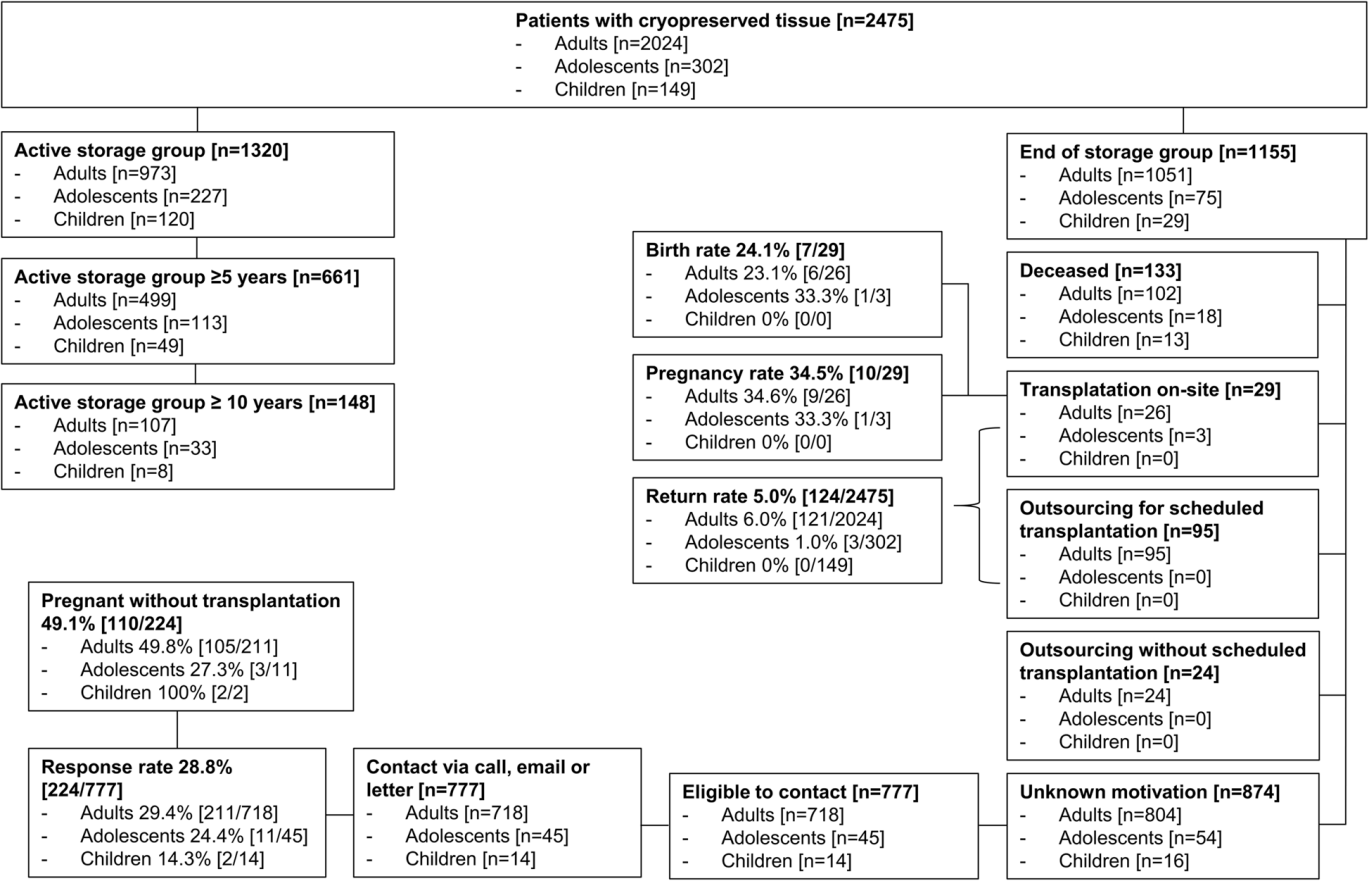
Flowchart of the study population and overall study design. Patient populations per storage group (n). Outsourcing: transportation of samples to another center. Inclusion criterion: female gender. Exclusion criteria: maler gender, distant metastases and significantly reduced probability of survival

This comprehensive analysis examined ovarian tissue samples from 2,475 patients undergoing OTC. Children (0–14 years) comprised 6% (149 patients) of the cohort, with a median age of 13 years (IQR 3), while adolescents (15–19 years) accounted for 12% (302 patients), with a median age of 17 years (IQR 1, Table 1). Within the pediatric group, 22.1% (33 patients) were under 10 years old, and 8.7% (13 patients) were younger than 5 years, with the youngest patient being only 3 months old at the time of OTC. The study population, including the active storage (AS) groups and the reasons for storage termination, is illustrated in Fig. 1.

**Table 1:** Ovarian tissue cryopreservation data of all patients, children, adolescents and adults

**Cryopreservation data**			**All indications**	**Haematological malignancies**	**Brain and nervous system**	**Sarcoma**	**Gynaecological tumours**	**Breast cancer**	**Lymphoma**	**Others**	**Non specified**
All patients	Patients	N	**2475**	**60**	**72**	**146**	**162**	**1108**	**555**	**207**	**165**
	Age [Years] at time of cryopreservation	Median Min-max IQR	**28** 0–44 6	**20** 3–38 5	**21.5** 2–39 4	**18** 5–36 3.8	**27** 8–43 7	**31** 19–44 2	**23** 12–41 4	**21** 0–40 6	**28** 0–40 7
	Tissue surface prior to cryopreservation [cm^2^]	Median Min-max IQR	**4.5** 0.5–25 1	**4.75** 0.6–19 2	**5** 1–15 1.5	**4.5** 0.5–17 1.5	**5** 1–22 1.5	**4.5** 0–25 1	**4.5** 0.5–20 1	**4.5** 0–20 1.5	**4.5** 0.75–16 1.5
All active storage patients	Active storage patients	N	**1320**	**38**	**48**	**88**	**78**	**496**	**353**	**124**	**95**
	Active storage patients ≥ 5 years	N	661	28	19	31	49	254	195	52	33
	Active storage patients ≥ 10 years	N	148	8	6	6	20	40	41	12	15
Children	Patients	N	**149**	**12**	**11**	**37**	**7**	**-**	**23**	**49**	**10**
	Age [Years] at time of cryopreservation	Median Min-max IQR	**13** 0–14 3	**11.5** 3–14 3.75	**10** 2–14 5	**13** 5–14 2	**13** 8–13 1	**-**	**14** 12–14 1	**11** 0–14 4	**11.5** 0–14 1.25
	Tissue surface prior to cryopreservation [cm^2^]	Median Min-max IQR	**3.5** 0.5–22 1.5	**2** 0.6–9 0.13	**3** 1.5–8 1.25	**3.5** 0.5–10.5 2	**7.25** 3.5–22 1.6	**-**	**5.5** 0.5–14 2.25	**3** 1–20 1	**4.5** ** 1.5–11 ** **2**
All active storage children	Active storage patients	N	**120**	**9**	**9**	**27**	**5**	**-**	**20**	**41**	**9**
	Active storage patients ≥ 5 years	N	49	7	1	11	3	-	10	15	2
	Active storage patients ≥ 10 years	N	8	2	-	2	1	-	-	2	1
Adolescents	Patients	N	**302**	**17**	**16**	**49**	**26**	**4**	**128**	**39**	**23**
	Age [Years] at time of cryopreservation	Median Min-max IQR	**17** 15–19 1	**17** 15–19 2	**18** 15–19 2	**17** 15–19 1	**17** 15–19 1	**19** 19-19 0	**18** 15–19 2	**16** 15–19 1	**18** 15–19 1
	Tissue surface prior to cryopreservation [cm^2^]	Median Min-max IQR	**4.5** 1–20 1	**8** 2.5–13 4	**6** 3–15 1.5	**5** 1–12.5 1.5	**5** 1.5–19 2.25	**5.75** 3.5–7.5 1.5	**4.5** 1–20 1.5	**4.5** 1.5–10 2	**4.5** 1–16 1
All active storage adolescents	Active storage patients	N	**227**	**14**	**13**	**34**	**17**	**3**	**100**	**30**	**16**
	Active storage patients ≥ 5 years	N	113	11	7	10	12	3	53	11	6
	Active storage patients ≥ 10 years	N	33	4	3	1	6	-	9	4	6
Adults	Patients	N	**2024**	**31**	**45**	**60**	**129**	**1104**	**404**	**119**	**132**
	Age [Years] at time of cryopreservation	Median Min-max IQR	**30** 20–44 5	**28** 20–38 5.5	**25** 20–39 3	**28** 20–39 2	**29** 20–43 4	**31** 20–44 2	**25** 20–41 3	**27** 20–40 3.5	**29** 20–40 3
	Tissue surface prior to cryopreservation [cm^2^]	Median Min-max IQR	**4.5** 0.5–25 1	**5.25** 1.5–19 1.87	**5.75** 1–12 2.25	**7** 1–12 3	**5** 1–25 1.5	**4.5** 0.5–25 1	**4.5** 1–15 1	**5** 0.5–18 1	**4.5** 0.75–16 1.5
All active storage adults	Active storage patients	N	**973**	**15**	**26**	**27**	**56**	**493**	**233**	**53**	**70**
	Active storage patients ≥ 5 years	N	499	10	11	10	34	251	132	26	25
	Active storage patients ≥ 10 years	N	107	2	3	3	13	40	32	6	8

*N* number, *min* minimum, *max* maximum, *IQR* interquartile range

**Fig. 2:**
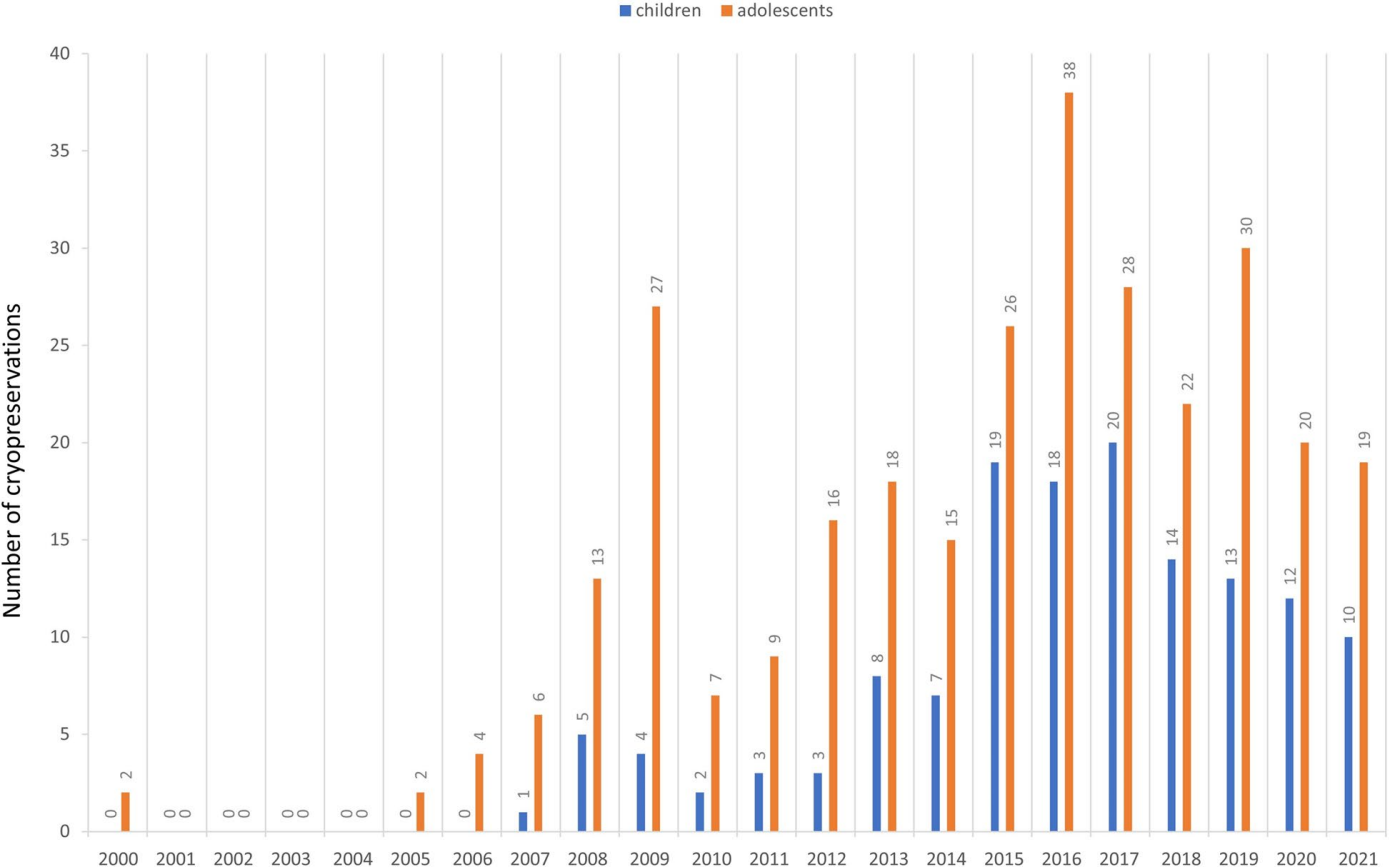
Annual number of ovarian tissue cryopreservation procedures performed in children and adolescents from 2000 to 2021, categorized by age groups. Total numer of cryopreservations in children *n* = 149. Total number of cryopreservations in adolescents *n* = 302

The annual number of OTC procedures performed in children and adolescents from 2000 to 2021 is illustrated in Fig. 2. The first OTC procedure in a child was conducted in 2007, after which cryopreservation rates steadily increased, reaching their peak between 2015 and 2017. In adolescents, OTC procedures were initiated earlier than in children, with a noticeable decrease observed toward the end of the first decade of the study period. This was followed by a gradual increase in procedures during the second decade.

### Age-dependent return rates, pregnancy rates and birth rates after OTT

The overall return rate of cryopreserved ovarian tissue was 5.0% (124 patients). Return rate was defined as either transplantation performed on-site (29 patients) or the transfer of tissue to an external center for planned transplantation (95 patients). In the group of children and adolescents, no cases of tissue outsourcing for scheduled or unscheduled transplantation to an external center were reported. The return rate among children was 0% (0 patients), while the return rate among adolescents was 1.0% (3 patients). The overall rate of at least one pregnancy following internal OTT was 34.5% (10 patients). Among adolescents, the pregnancy rate was 33.3%, with one patient achieving pregnancy and currently experiencing a second ongoing pregnancy. A total of 24.1% (7 patients) of all individuals who underwent internal OTT successfully gave birth at least one time, including 33.3% (1 patient) in the adolescent group. Notably, three patients achieved two pregnancies from the same OTT. One patient who gave birth following oocyte donation and another who conceived using the existing ovary rather than the transplanted tissue were excluded from these calculations.

### Age-dependent distribution in the active storage group

In total, 53.3% (1,320 patients) of all patients had ongoing AS. Within this group, children accounted for 9% (120 patients) with a median age of 12 years (IQR 2) at OTC and a median storage duration of 4.2 years (IQR 1.7), with a maximum storage duration of 14.1 years. Adolescents represented 17% (227 patients) of the AS group, with a median age of 17 years (IQR 1) at OTC and a median storage duration of 4.9 years (IQR 2.3), with a maximum storage duration of 15.2 years.

The distribution of AS across different malignancies varied. In hematological malignancies, 23.7% (9 patients) were children and 36.8% (14 patients) were adolescents. For brain and nervous system tumors, children represented 18.8% (9 patients) and adolescents 27.1% (13 patients). In sarcomas, children accounted for 30.7% (27 patients) and adolescents for 38.6% (34 patients). Gynecological tumors showed 6.4% (5 patients) children and 21.8% (17 patients) adolescents. Breast cancer AS was absent in children and present in only 0.6% (3 patients) of adolescents. Lymphoma AS included 5.7% (20 patients) children and 28.3% (100 patients) adolescents. Other indications comprised 33.1% (41 patients) children and 24.2% (30 patients) adolescents. Non-specified cases included 9.5% (9 patients) children and 16.8% (16 patients) adolescents.

**Fig. 3:**
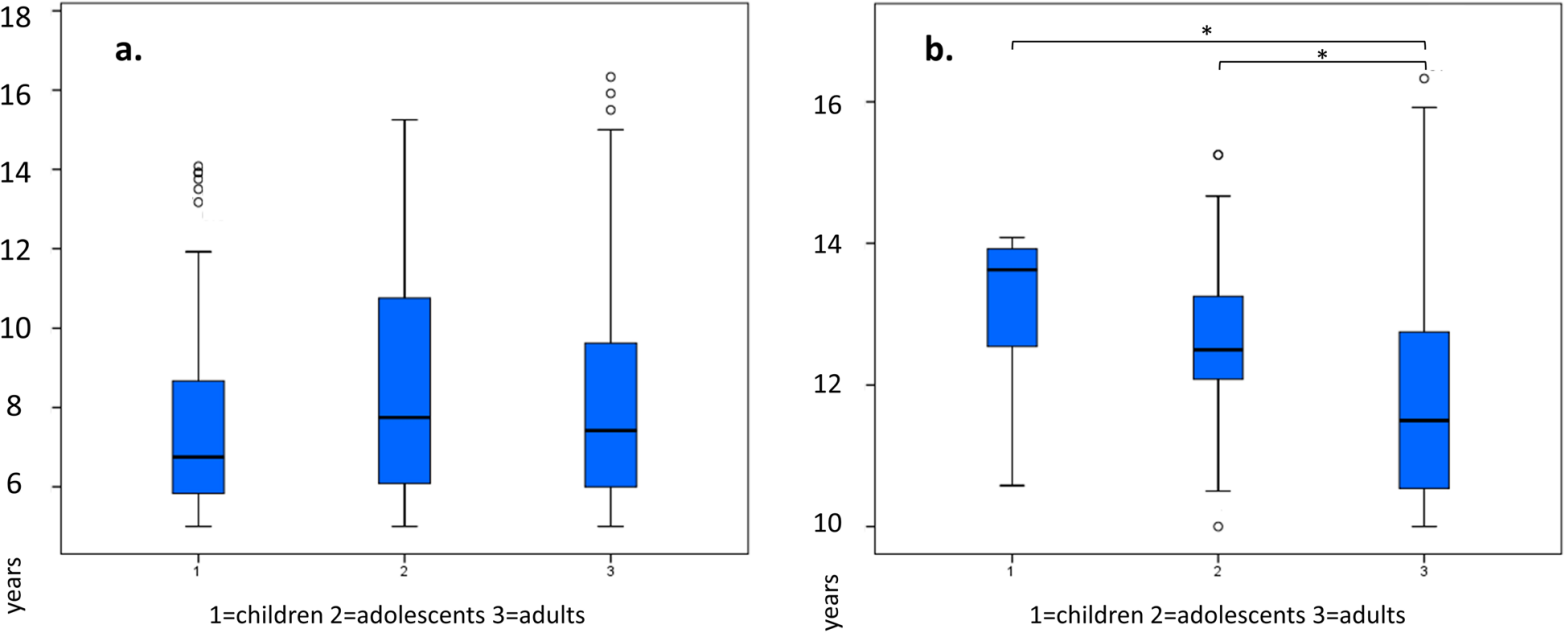
Comparison of active storage duration in different age groups in. **a** active storage group ≥ 5 years in years with total number of patients *n* = 661. **b** active storage group ≥ 10 years in years with total number of patients *n* = 148. *asterisk indicating statistical significance p ≤ 0.01

Subgroups of AS duration were also analyzed: 50.7% (661 patients) had AS ≥ 5 years, and 11.2% (148 patients) had AS ≥ 10 years. Age-dependent differences in storage duration were observed across various active storage (AS) groups. In the overall AS group, adults demonstrated significantly longer storage times compared to children (mean 5.5 years, 95% CI 5.3–5.7 vs. 4.7 years, CI 4.2–5.3; *p* = 0.011). However, this trend reversed in the AS group ≥ 10 years, where children stored significantly longer than adults (mean 13.1 years, CI 12.1–14.1 vs. 11.8 years, CI 11.5–12.1; *p* = 0.01). Similarly, adolescents in the AS group ≥ 10 years also stored significantly longer than adults (mean 12.6 years, CI 12.1–13.1 vs. 11.8 years, CI 11.5–12.1; *p* = 0.002). No significant differences were found between age groups in the AS ≥ 5 years category (Fig. 3).

**Fig. 4:**
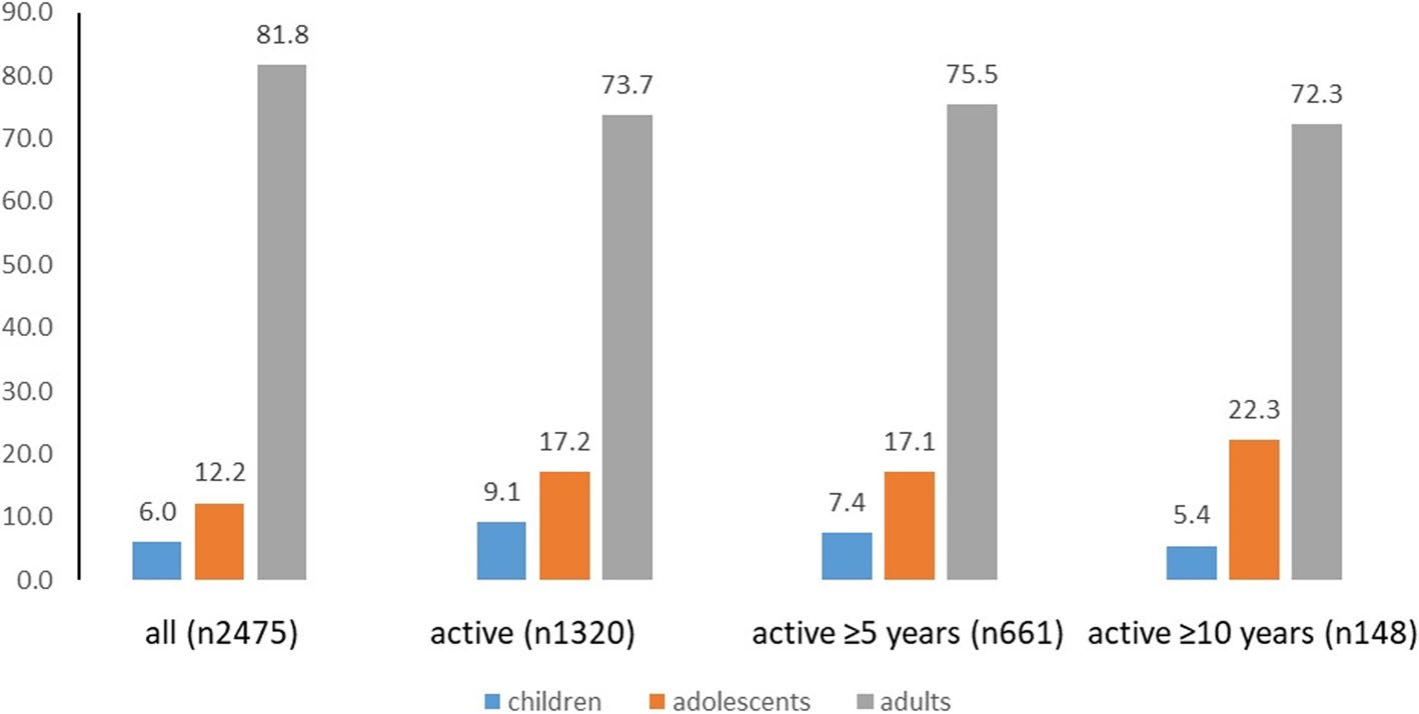
Age compositions at time of OTC and during storage in percent of all patients, patients with active storage (AS), in the group of AS ≥ 5 years and AS ≥ 10 years. *n* = number of patients

Analysis of age compositions at the time of OTC and over time revealed an increasing proportion of adolescents and a decreasing number of children in the long-term storage group (Fig. 4).

### Diagnostic distribution in pediatric and adolescent ovarian tissue cryopreservation

The most prevalent malignancies differed between age groups, with sarcoma being predominant in children (37 patients) and lymphoma in adolescents (128 patients). The distribution of patients across various disease categories revealed distinct patterns. In hematological diseases, children represented 20% (12 patients) and adolescents 28.3% (17 patients) of cases. For brain and nervous system tumors, children accounted for 15.3% (11 patients) and adolescents 22.2% (16 patients). Sarcomas showed a prevalence of 25.3% (37 patients) in children and 33.6% (49 patients) in adolescents.

**Fig. 5:**
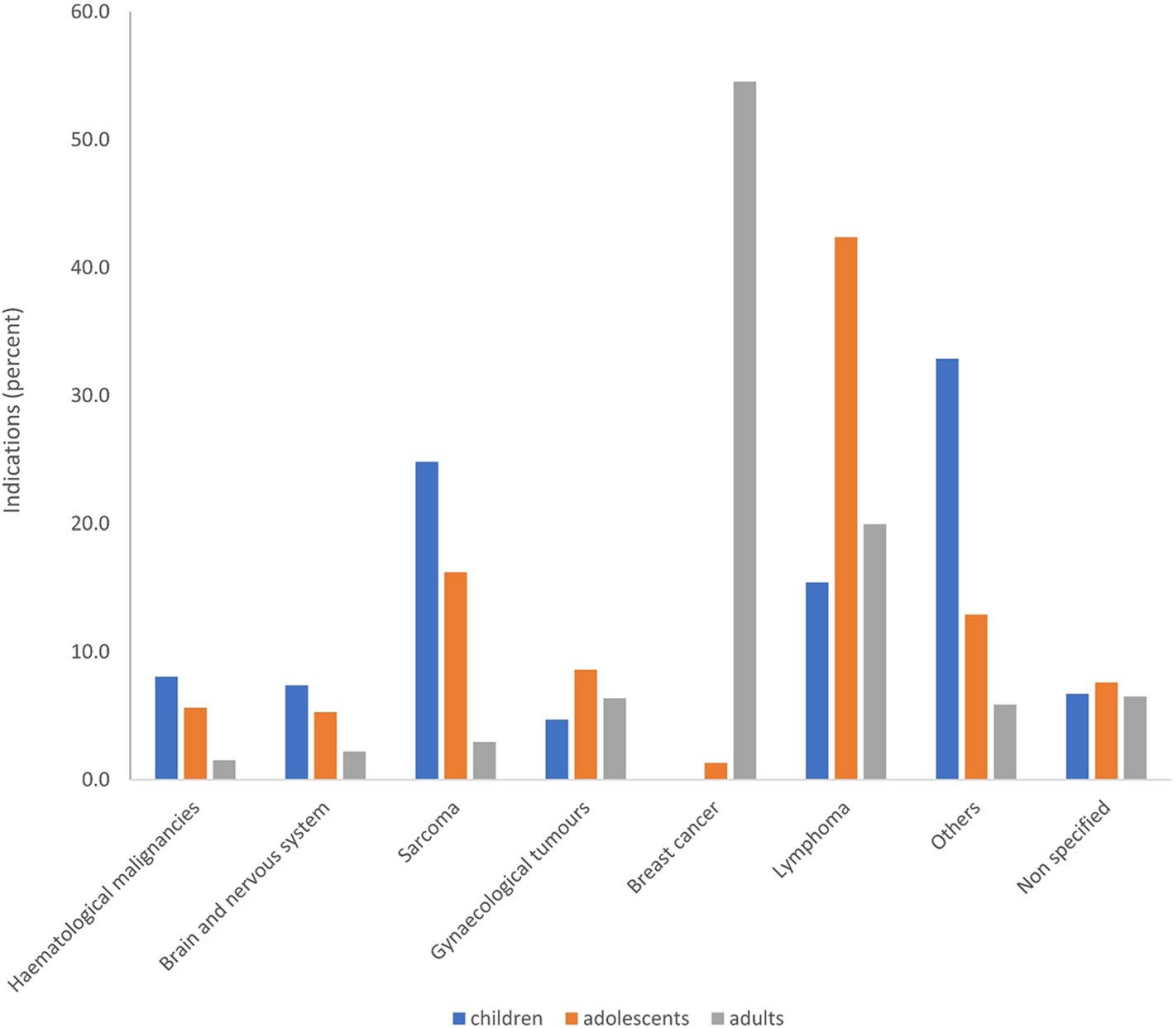
Age related indications of all patients with OTC in percent with total number of patients *n* = 2,475

**Fig. 6:**
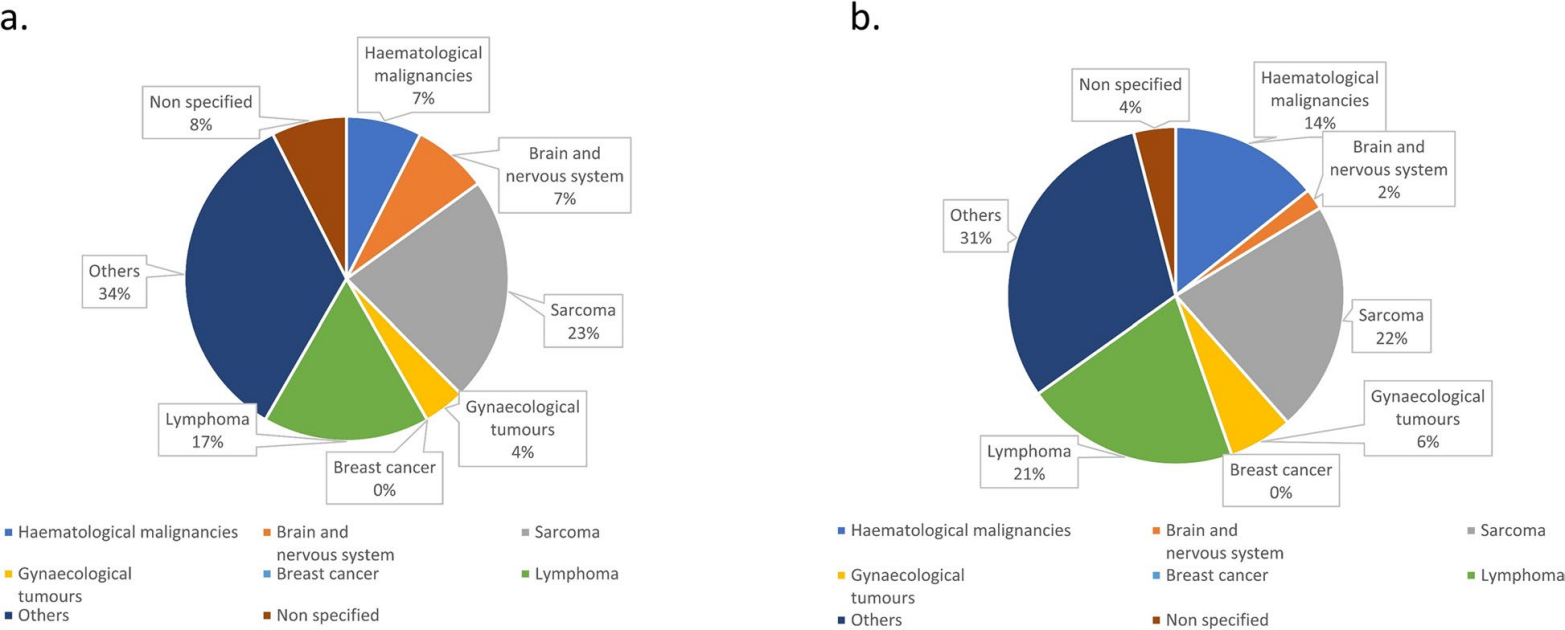
Distribution of indications in children in. **a** active storage group in percent with total number of patients *n* = 120. **b** active storage group ≥ 5 years in percent with total number of patients *n* = 49

Gynecological tumors were observed in 4.3% (7 patients) of children compared to 16% (26 patients) of adolescents. Breast cancer was absent in children and present in 0.4% (4 patients) of adolescents. Lymphomas were found in 4.1% (23 patients) of children and 23.1% (128 patients) of adolescents. Other malignancies accounted for 23.6% (49 patients) in children and 18.8% (39 patients) in adolescents. Unspecified cases represented 6% (10 patients) in children and 13.9% (23 patients) in adolescents (Table 1, Figs. 5 and 6).

### Mortality rates

Between 2000 and 2021, 5.4% (133 patients) of all individuals who stored ovarian tissue at the University Hospital Bonn's cryobank passed away. Among children, the mortality rate was 8.7% (13 patients), while 6.0% (18 patients) of adolescents died during this period. Mortality rates varied significantly based on tumor type in children who underwent OTC. In the hematological malignancies group, 25% (3 patients) died. For brain and nervous system tumors, the mortality rate was 9% (1 patient), and for sarcomas, it was 13.5% (5 patients). No deaths were recorded among children with gynecological tumors, while 4.3% (1 patient) with lymphoma passed away. Similarly, mortality rates among adolescents who underwent OTC differed by tumor type. In the hematological malignancies group, 5.9% (1 patient) died. For brain and nervous system tumors, the mortality rate was 12.5% (2 patients), and for sarcomas, it was 10.2% (5 patients). In the gynecological tumor group, 11.5% (3 patients) died, while no deaths occurred among adolescents with breast cancer. In the lymphoma group, the mortality rate was 0.8% (1 patient). Overall, children and adolescents accounted for 9.8% and 13.5%, respectively, of all deceased patients in this study cohort.

### Other reasons for storage ending

Children accounted for 2.5% (29 patients) and adolescents for 6.5% (75 patients) of the total group that discontinued storage. Beyond the previously described cases of death, transplantation, and outsourcing without scheduled transplantation, the motivations for ending storage in other cases were further investigated. Among the 14 children eligible for contact, two responded to outreach efforts. Of those patients, 100% reported the occurrence of a pregnancy without retransplantation of the ovarian tissue (2 patients). For the first patient, the indication for cryopreservation was osteosarcoma with scheduled chemotherapy. The second patient presented with a torsioned right ovary, showing interspersed fibroma, while the left ovary had multiple ovarian fibromas. Given the bilateral ovarian tumors with uncertain malignancy, a right adnexectomy and partial left ovarian resection were performed, along with cryopreservation of left ovarian tissue.

Of the 45 adolescents contacted, 24.4% provided responses (11 patients). Of those, 27.3% reported pregnancies without retransplantation (3 patients). Notably, all three of these patients had a history of Hodgkin's lymphoma. Furthermore, 18.2% expressed no desire to have children (2 patients), and another 18.2% experienced a recurrence of cancer (2 patients). 9.0% discontinued storage due to financial constraints (1 patient), 36.4% passed away (4 patients), and 27.3% did not provide reasons for ending storage (3 patients).

Among the 63 patients under the age of 18, 8 discontinued storage upon reaching adulthood. In 5 cases, this was due to the patient’s death, while in the remaining 3 cases, it resulted from the voluntary destruction of the cryopreserved tissue. Thus, reaching the age of majority did not appear to influence the decision to terminate storage duration.

### Tissue surface prior to cryopreservation

In the pediatric cohort, the median surface area of ovarian cortex tissue prior to cryopreservation was 3.5 cm^2^ (range 0.5–22, IQR 1.5), while in the adolescent cohort it was 4.5 cm^2^ (range 1–20, IQR 1).

Among children, patients with gynecological tumors had the largest tissue surface area, with a median of 7.25 cm^2^ (range 3.5–22, IQR 1.6).

In the adolescent group, patients with hematological malignancies had the largest cryopreserved tissue surface area, with a median of 8 cm^2^ (range 2.5–13, IQR 4). This was followed by patients with breast cancer and gynecological tumors, both with median surface areas of 5.75 cm^2^ (range 3.5–7.5, IQR 1.5) and 5 cm^2^ (range 1.5–19, IQR 2.25), respectively (Table 1).

## Discussion

This study comprehensively examined the distribution of indications for OTC in children and adolescents and analyzed age-dependent storage behaviors. With 451 patients, this represents the largest single-center study investigating OTC in pediatric populations to date [41–49].

### Current research landscape: OTC recommendations for pediatric and adolescent patients

The American Society of Clinical Oncology and German guidelines strongly recommend counseling pediatric patients and their parents before gonadotoxic treatment due to the high risk of sterility from chemotherapy [8, 47]. OTC is the only viable fertility preservation option for prepubertal girls and has become standard practice in several countries, although it is still classified as investigational in children [19, 21, 50]. A systematic review reported ten live births and one ongoing pregnancy from 18 patients who underwent OTC before age 21, with the youngest successful case being a 9-year-old girl [24, 51–60]. While OTC can restore ovarian function and enable pregnancies in girls aged 9 and older, outcomes for younger children require further study [24, 56, 60–66]. In a study by Jensen et al., 71.8% of prepubescent girls who underwent OTC needed medical puberty induction, highlighting the need for yearly follow-ups with hormone analysis during puberty [15, 48]. Although two cases of OTT in prepubertal girls successfully induced puberty [67–69], concerns about its impact on accelerated development and psychological distress favor medically induced puberty using low-dose hormones. The required amount of ovarian tissue for achieving pregnancy remains unknown, necessitating careful consideration of its use since cryopreserved oocytes are limited [66, 70, 71].

Decision-making for OTC should consider the risk of premature ovarian insufficiency (POI), which is expected to be at least 20–50% in patients undergoing moderate to high-risk treatments [30, 66, 72]. Additionally, sufficient egg reserve at the time of OTC is crucial for future pregnancies, with AMH levels recommended to be above 0.5 ng/ml [72], but this should be interpreted with caution in children and adolescents until the age of 20 years, as AMH and follicle density do not necessarily correlate in this group of patients [73]. Duffin et al. showed that the Edinburgh Selection Criteria effectively identify 12- to 18-year-old girls at risk of POI, aiding OTC eligibility decisions [74]. Laparoscopic ovarian tissue removal has a low complication rate (0.2–0.6%) [75–77] and recent studies show no increase in childhood cancer or congenital malformations among offspring of cancer survivors [78].

### Global comparison of return, pregnancy, and birth rates

The return rate of 5.0% observed in this study is consistent with other reports, such as Jadoul et al. (4.4%), Van der Ven et al. (3%), Jensen et al. (5%), Diaz et al. (6.2%), and Lotz et al. (3.6%) [53, 79–82]. However, none of these studies provide specific return rate data for the subgroups of children or adolescents. In the current study, the return rate for ovarian tissue was 0% among children and 1.0% among adolescents. Additionally, this study highlights an unexpected finding: notably high pregnancy rates were observed without OTT, with rates of 100% (2 patients) in children, 27.3% (3 patients) in adolescents, and 49.8% (105 patients) in adults. These results provide novel insights into fertility outcomes in this young population. The observed low utilization rates of ovarian tissue cryopreservation and high pregnancy rates without OTT highlight a critical need for enhanced understanding of premature ovarian insufficiency risks following gonadotoxic treatments. By developing a more comprehensive understanding of individual risk factors, healthcare providers can offer this fertility preservation technique more selectively and effectively. This targeted approach would optimize resource allocation, improve patient outcomes, and align with the principles of personalized medicine in oncofertility care.

The pregnancy and birth rates following OTC/OTT in this study – 34.5% and 24.1% in adults, and 33.3% in adolescents – rank among the highest in international comparisons. For example, Van der Ven et al. reported pregnancy and birth rates of 32.7% and 30.3%, respectively, with no pregnancies achieved by the single adolescent included [53]. Meirow et al. observed a 50% pregnancy rate and 30% birth rate, though none of the four adolescents undergoing OTC achieved pregnancy [59]. Jadoul et al. documented 33.3% pregnancy and birth rates after OTT but provided no data specific to adolescents [82]. Diaz-Garcia reported rates of 27.3% for pregnancies and 18.2% for births, while Gellert et al. noted 25.4% and 18%, respectively, with neither study offering pediatric-specific data [79, 83]. Fortin et al. published pregnancy and birth rates of 29.4%, Dittrich et al. reported 35.0% and 20.0%, and Donnez and Dolmans documented a birth rate of 42.1%, but none included children or adolescents [84–86]. Hoekman et al. observed a combined pregnancy and birth rate of 57.1% in four patients, while Takae et al. reported rates of 33.3% for pregnancies and 11.1% for live births, with no patients under 20 years old in either study [87, 88]. These findings emphasize the need for further research into fertility outcomes specific to younger populations undergoing OTC/OTT.

### Interpretation of age-dependent storage durations of ovarian tissue

To our knowledge, no data on storage duration in OTC for children and adolescents have been published so far. Among those with storage durations of ≥ 10 years, children and adolescents show a significantly higher proportion compared to adults. This may be due to the longer time required for them to reach reproductive age and consider starting a family, as well as the higher prevalence of hematologic malignancies in this group, leading to extended waiting periods for IVG/IVM. Consistently, the proportion of pediatric patients with hematologic malignancies and sarcomas increases with longer storage durations. For conditions like leukemia or Ewing sarcoma, where the risk of retransferring malignant cells through OTT is significant, IVG/IVM remains the only safe fertility option to prevent cancer recurrence after transplantation [28, 29, 87–91].

Interestingly, within the AS group as a whole, adults stored tissue for significantly longer periods than children. At the same time, the proportion of adolescents with storage durations of ≥ 10 years was increasing, while the number of younger children in this category was declining. This shift may reflect the limited use of OTC in younger children prior to 2010, when experience with the procedure in this age group was still developing. Additionally, the higher mortality rate in children (8.7% compared to 6.0% in adolescents) may further explain this pattern.

These findings are relevant, as extended storage durations pose a substantial financial burden on patients and their families. Furthermore, these observations not only shed light on evolving practices in pediatric fertility preservation but also highlight the importance of refining counseling strategies to address the unique challenges and long-term considerations for younger patients.

### Interpretation of age-dependent indications for OTC

The most common cancer types in children and adolescents are leukemia, central nervous system neoplasms, lymphoma, and malignant bone tumors [1]. This distribution is reflected in OTC indications across multiple studies. Our cryobank data aligns with this pattern, showing sarcoma, lymphoma, hematological malignancies, and brain tumors as primary indications. Similar findings are reported in various international studies, with slight variations due to regional factors and specific center collaborations [4, 41–43, 45, 46, 89–92]. For instance, some centers report higher rates of hemoglobinopathies due to partnerships with sickle cell disorder experts, while others show increased prevalence of Epstein-Barr virus infections in certain populations. Despite these minor differences, the overall indications for OTC consistently mirror the most frequent pediatric cancer types, emphasizing the importance of fertility preservation in young patients undergoing gonadotoxic treatments. The proportion of children and adolescents undergoing OTC is small. This analysis of the distribution of indications is important to determine whether there is a particularly large care gap in any specific medical specialty. It appears that the proportion of patients seeking fertility preservation counseling is comparable to the distribution of primary diseases in children and adolescents. Therefore, it can be assumed that there is no specific medical specialty with a particularly large care gap in this regard. These findings demonstrate the diverse range of indications for OTC in pediatric and adolescent populations, underscoring the importance of fertility preservation across various oncological conditions.

### Comparison of mortality rates

This study observed mortality rates of 8.7% and 6.0% in children and adolescent groups, respectively, which are lower than those reported in several other studies. Published mortality rates for children undergoing ovarian tissue cryopreservation (OTC) range from 5% to 27.8% [47, 89], with most studies reporting rates between 6.6% and 21.6% [41, 42, 45, 48, 49, 90, 91]. The highest relative mortality in this study was observed in hematological malignancies (25%, 3 patients) and sarcomas (13.5%, 5 patients), aligning with findings from other studies that consistently report elevated mortality rates in these disease categories. Fabbri et al. reported highest mortality in sarcomas (38%) and hematological diseases (30%) [41], while Lotz et al. noted highest rates in leukemia (25%) and sarcoma (23.5%) [90]. Similarly, Poirot et al. identified sarcoma (28.2%) and leukemia (20%) among the top five OTC indications with highest mortality rates [42].

While these findings could contribute to more nuanced risk–benefit assessments for OTC in pediatric patients with sarcoma and leukemia, it's crucial to interpret the results cautiously due to the small sample size. Nevertheless, these findings might help refine counseling approaches, potentially leading to more tailored treatment decisions that consider the observed mortality trends in these specific patient groups, thereby potentially reducing instances of overtreatment. The low return rates as well as favorable pregnancy rates observed in children and adolescents without OTT in this study further support the notion that OTC may, in some cases, be overutilized.

### International comparison of OTC age demographics

In this study, the median age of children undergoing OTC was 13 years (range 0–14). Other German studies reported mean ages of 14.8 years (range 6–17) and 14 years (range 1–17) [46, 90]. In contrast, French studies found a median age of 6.9 years (range 0.3–15) and a mean age of 9.3 years (range 0.2–17) [42, 45]. In China, the mean age was 7.55 ± 3.64 years (range 1–14) [43], while in the USA, the median age was 12 years (range 5 months–23 years) [91]. In Denmark, the mean age was 8.11 years for children aged ≤ 12 and 11.25 years overall (range 0.6–17.11) [48]. Belgium reported a mean age of 10.3 years (range 0.8–15.8) [49], and an Italian study found a mean age of 12.9 years in patients ≤ 17 years [41]. Notably, countries where OTC is covered by healthcare systems – such as France, Denmark, Israel, Spain, and Belgium [93, 94] – tend to have younger average ages for OTC compared to countries like China, the UK, and most states of the USA, where patients often bear the costs [95, 96]. Germany recently decided to cover OTC costs through its healthcare system; however, during this study’s period, patients paid out-of-pocket [97]. Remarkably, the average age for OTC in children and adolescents in Germany is significantly higher than the international average [46, 90]. Internationally, average fees for ovarian tissue removal, cryopreservation with five years of storage, and transplantation amount to €5,000, €4,000, and €5,000 respectively [93]. Since children typically require longer storage durations, costs are even higher. Financial barriers are significant: In this study, 9.0% of the adolescents terminated storage due to financial reasons. Piselli et al. reported tissue discard in 10.8% of cases due to missed payments and financial concerns in 29.7% of patients [98], while Schallmoser et al. found that 8.9% ended storage due to high costs [35]. These findings stress the urgent need for healthcare funding for OTC, especially in children. Beyond financial factors, demographic differences across countries may also be influenced by limited awareness among pediatric oncologists and/or an underdeveloped referral and supply network for ovarian tissue cryopreservation. These findings highlight disparities in OTC practices globally due to differences in healthcare systems and demographics. Further research is needed to address these challenges and improve access to fertility preservation for children worldwide.

### Average surface area of cryopreserved ovarian cortex

For adults, it is recommended to cryopreserve approximately half of an ovary. Dolmans et al. suggest performing unilateral oophorectomy in young girls because of the small size of children’s ovaries, but so far there is no common consensus on this issue [66]. Current evidence suggests that unilateral oophorectomy brings forward menopause by one to two years [66, 99, 100].

In this present study, the majority of children and young adolescents underwent unilateral oophorectomy. Median surface of ovarian cortex in children (0–14 years) was 3.5 cm^2^ and 4.5 cm^2^ in adolescents (15–19 years). These results are in line with current literature. In a study by Ruan et al., mean cryopreserved surface of the ovarian cortex in children (1–14 years) was 3.7cm^2^ [43]. Grellet-Grün et al. published mean surface of 2.6 cm^2^ in the group of children and adolescents (0.2–17 years) [45]. In the cohort of children ≤ 14 years by Jensen et al., mean surface of cryopreserved ovarian cortex was 3.15 cm^2^ and in the cohort of adolescents (15–18 years), mean surface was described as 5.5 cm^2^ [48]. Abovementioned authors also performed laparoscopic unilateral ovarectomy in children due to small ovary sizes [43, 43, 45, 48]. These findings could contribute to a more joint consensus regarding the necessary amount of harvested ovarian tissue for OTC in children and adolescents.

To our knowledge, this is the first study to examine ovarian tissue distribution across different OTC indications in children. The high proportion of large cryopreserved tissue in gynecological tumors may stem from bilateral ovary removal in some cases. For breast cancer patients, chemotherapy often includes cyclophosphamide, a highly gonadotoxic agent due to its oxidative stress and DNA methylation effects [19], possibly prompting greater ovarian tissue removal. Similarly, high-dose busulfan used before stem cell transplantation in hematologic malignancies is highly damaging to ovarian tissue [19], likely leading to increased tissue excision in these pediatric patients. Understanding the distribution of ovarian tissue across different indications can help refine surgical and preservation techniques, ensuring better outcomes for pediatric patients in terms of fertility restoration and overall health.

## Limitations

It is important to note that this study has limitations, particularly as it is a retrospective analysis. Consequently, information regarding the stage of cancer and initial chemotherapy prior to OTC was not available. Pregnancy and birth rates in adolescents should be interpreted with caution due to the low retransplantation rate within this age group. Additionally, pregnancy rates without retransplantation should also be viewed cautiously in the groups of children and adolescents, as they are based on small sample sizes and may be influenced by potential publication bias. This bias arises from the likelihood that patients are more inclined to respond to contact attempts when they have positive outcomes, such as pregnancies, to report. Furthermore, the return rates observed in children and adolescents should be interpreted with caution, as longer observation periods – potentially exceeding two decades – may be necessary to draw more definitive conclusions on this subject. Moreover, caution is warranted when interpreting mortality rates due to the limited sample sizes, which may affect the generalizability of the findings, highlighting the need for larger studies to validate these results. Additionally, the current study does not include a cost–benefit analysis; therefore, any discussion regarding costs should be approached with caution. Furthermore, the lack of detailed information regarding the type and quality of fertility counseling provided to patients at external centers should be mentioned. This absence of data prevents an assessment of whether the counseling was comprehensive or superficial. Consequently, this limitation may result in a restricted understanding of the factors influencing patient choices and a lack of critical contextual information regarding their decision-making processes.

## Conclusions

OTC is a viable and low-risk option for fertility preservation in children and adolescents, with a high potential for successful pregnancies [18, 19, 52, 97, 101]. Indications for OTC align with common pediatric malignancies [4]. Notably, children and adolescents tend to store ovarian tissue for significantly longer durations than adults in the group of AS > 10 years, likely due to their delayed progression to reproductive maturity and longer waiting times for fertility treatments like IVG/IVM in cases of hematological diseases. Conversely, adults generally have longer storage periods overall and adolescent storage has risen over time in comparison to children, which may be influenced by the higher mortality rates among children and the historically limited experience with OTC in this younger demographic prior to 2010.

The findings regarding the mean surface area of cryopreserved ovarian cortex in pediatric patients may help establish a consensus on the optimal amount of harvested tissue, with unilateral oophorectomy recommended. Despite the small proportion of children and adolescents undergoing OTC compared to adults, discussions about fertility preservation should be integral to standard care for pediatric patients facing gonadotoxic treatments. Timely referrals to fertility specialists are essential to preserve reproductive autonomy. Additionally, this study suggests a potential correlation between the costs associated with OTC and its implementation, highlighting the need for cost coverage in healthcare systems to support affected patients.

A tailored and individualized approach to determining the appropriate indications for OTC in children and adolescents is essential, particularly in cancer subgroups such as leukemia or sarcoma, where mortality rates remain relatively high. Moreover, the favorable pregnancy rates observed in this study among children and adolescents without retransplantation of ovarian tissue further underscore the need for careful consideration, as these findings suggest that OTC may, in some cases, be overutilized.

The findings of this study emphasize the importance of individualized fertility preservation strategies and careful clinical decision-making to mitigate long-term risks while preserving reproductive potential.

## Data Availability

The datasets used and/or analyzed during the current study are available from the corresponding author on reasonable request.

## Abbreviations

AS: Active storage
GnRHa: Gonadotropin-releasing hormone agonists
IQR: Interquartile range
OTC: Ovarian tissue cryopreservation
OTT: Ovarian tissue transplantation
